# Imprinted *Xlr3* (*X-linked Lymphocyte Regulated 3*) produces a meiosis specific protein implicated in sex chromosome gene regulation in mouse

**DOI:** 10.1186/1756-8935-6-S1-P19

**Published:** 2013-03-18

**Authors:** Robert J Foley, Seth Kasowitz, Emily Clancy, Andrew Sadowski, Michael O’Neill

**Affiliations:** 1 Department of Molecular and Cell Biology, University of Connecticut, Storrs, CT, 06066, USA; 2 Department of Animal Biology, University of Pennsylvania School of Veterinary Medicine, Philadelphia, PA, 19104, USA

## Background

Meiotic crossingover is an important checkpoint in almost all sexually reproducing eukaryotic organisms and is mediated by the Synaptonemal Complex (SC) during meiosis I. In this process homologous chromosomes exchange sequence information before segregating [1]. Some proteins involved in this process (i.e - SYCP3) contain a conserved COR1 domain of which the function is not known, however; these proteins tend to be present only in germ cells [1-4].*Xlr3* (*X-linked lymphocyte regulated 3*), an X-linked imprinted gene in mouse, also contains a COR1 domain but has yet to be characterized.

## Materials and methods

Protein and total RNA were isolated from testis sampled daily during the first wave of spermatogenesis. Gene expression was analyzed by real-time qRTPCR. Cell surface spreads were prepared by standard methods and used for immunohistochemistry.[4] Similar methods were used with embryonic ovary samples

## Results

mRNA analysis shows elevated relative expression of *Xlr3* transcripts in gonads where it becomes upregulated as the germ cells enter meiosis I. Translational analysis of XLR3 reveals the existence of a protein which is first detectable during the initiation of meiosis I. Visually, XLR3 localizes to the inactive X and Y chromosome during the pachytene stage of spermatogenesis. XLR3 localizes similarly at the sex chromosomes in female meiosis. Additionally, a second transient localization of XLR3 is seen at the post-meiotic repressed sex chromosomes (PMSC) in haploid round spermatids.

## Conclusions

*Xlr3* mRNA upregulation at the start of meiosis is consistent with initial presence of the XLR3 protein in early meiotic stage cells. Visually, XLR3 appears to localize to the inactive X and Y chromosome during meiosis I in males, and later to the repressed X or Y chromosomes after meiosis. This germ cell specific role is consistent with the localization of other COR1 domain proteins (i.e - SYCP3, SLY, SLX). These data, taken with the unique pairing of the X and Y at meiosis, may suggest a necessary gene regulatory role of XLR3 and other COR1 domain proteins on the sex chromosomes during and after meiosis in mice.
